# 45. A rare case of macrophage activation syndrome (MAS) associated with hypomyopathic dermatomyositis

**DOI:** 10.1093/rap/rky034.008

**Published:** 2018-09-20

**Authors:** Liubov Borukhson, Thushyanthan Guruparan, Sundeept Bhalara

**Affiliations:** Rheumatology, Watford General Hospital, Watford, UNITED KINGDOM

**Introduction:** Haemophagocytic lymphohistiocytosis (HLH) is a rare multi-system inflammatory condition, which represents a challenging diagnostic conundrum. Macrophage activation syndrome (MAS) is a term used to describe secondary HLH found in conjunction with underlying rheumatological diseases such as juvenile idiopathic arthritis, systemic lupus erythematosus, adult onset Still’s disease, periodic fevers, or Kawasaki disease. If left untreated, irreversible organ damage can ensue; the condition is lethal in up to 53% of cases. Recognition and treatment of the underlying trigger is crucial in controlling MAS. We present a very rare case of MAS associated hypomyopathic dermatomyositis (DM). This has rarely been reported in the literature in the past.

**Case description:** A 67 year old male with type one diabetes mellitus, controlled by insulin, presented with a three week history of a widespread erythematous rash, predominantly affecting the neck, chest, and back. He had progressive lethargy and fevers of up to 40 degrees C. As his disease progressed, he developed signs of encephalopathy with dizziness, lethargy and disorientation. He was pancytopenic with Hb - 111 g/L, WCC - 1.1x10^9^ /L, and platelets of 92x10^9^ /L. Liver enzymes were deranged with ALT of 310 U/L, ALP 69 U/L, and GGT 256 U/L. The striking feature was a profound hyperferritinaemia with a serum ferritin of 41,385 micrograms/L. There was a paradoxically low inflammatory response (CRP - 6.8 mg/L and ESR - 29 mm/min with normal values of TAG 1.20 mmol/L and fibrinogen level of 2.76 g/L. He did not report any muscle weakness and the creatinine kinase level was only mildly elevated at 350 units/L. The autoimmune screen revealed a weekly positive ANA (speckled pattern >1/100), anti-Ro positive, however negative Anti-La/DsDNA/ANCA/antiJo-1/antiSCL-70, antiRNP/antiSM and extended myositis antibody profile. The serum C3 and C4 level was normal. Extended viral and bacterial screening also revealed negative results. CT abdomen and pelvis was normal. Given the pancytopenia, recurrent fevers and a very high ferritin, MAS was suspected. Bone marrow trephines showed increased number of monocytes (5-10%), CD68+ with possible evidence of haemophagocytosis and trilineage dysplasia. Skin punch biopsy taken from upper back showed large areas of full thickness epidermal necrosis. Viable epidermis at the edges showed keratinocyte apoptosis. Although those findings were more typical of toxic epidermal necrolysis (TEN), cases of full thickness vesiculation have been described in DM. MAS was diagnosed on the basis of the Ravelli criteria 2016 defined in a febrile patient with juvenile arthritis of high ferritin of > 684 ng/mL and any two of the following: platelet count ≤181x10^9^/L; aspartate aminotransferase >48 units/L; triglycerides >156 mg/dL; or fibrinogen ≤360 mg/dL. The patient was initially treated with IV antibiotics, antivirals and antifungals in view of the pyrexia of unknown origin. Following the diagnosis of MAS, three pulses of 500mg methylprednisolone IV were administered, followed by a regime of 20mg dexamethasone daily and G-CSF. However, the patient continued to deteriorate and was admitted to ITU. He was intubated for respiratory distress, required haemofiltration due to severe AKI. Ciclosporin 175 mg twice daily, Anakinra 10mg daily and Intravenous immunoglobulin (IVIg) were added with a dramatic response and resolution of the pancytopenia and rash. Following this, maintenance methotrexate was added and the steroids were tapered. The ciclosporin was withdrawn. To date he remains in remission.

**Discussion:** We report a patient presenting with several features of MAS including pancytopenia, high ferritin and deranged LFTs, high fevers, encephalopathy and a bone marrow biopsy suggestive of hyperactivity of the monocyte/macrophage lineage. His rash was likely related to DM. As underlying pathogenesis in MAS is thought to be related to T-cell over-activation, we think in our case it was triggered by hypomyopathic DM. As with previous case reports of MAS associated with DM, our patient was steroid resistant. However, he responded well to a combination of ciclosporin, Anakinra and IVIg. He currently remains in remission on maintenance treatment with methotrexate and low dose corticosteroid.

**Key Learning Points:** Despite the rarity of MAS, it is a diagnosis to be remembered in an obtunded patient who presents with combination of cytopenia, fever, encephalopathy and high serum ferritin level. Amyopathic or hypomyopathic DM can be triggering disease and all patients should be carefully assessed for rashes. The diagnosis and management are difficult and need close collaboration between rheumatology, haematology and intense care teams. A favourable treatment response was achieved with aggressive and prompt immunosuppression using a combination of corticosteroids, methotrexate, ciclosporin, anakinra and IVIg.

**Disclosure: L. Borukhson:** None. **T. Guruparan:** None. **S. Bhalara:** None.

